# Coherent detection-based photonic radar for autonomous vehicles under diverse weather conditions

**DOI:** 10.1371/journal.pone.0259438

**Published:** 2021-11-15

**Authors:** Sushank Chaudhary, Lunchakorn Wuttisittikulkij, Muhammad Saadi, Abhishek Sharma, Sattam Al Otaibi, Jamel Nebhen, Demostenes Zegarra Rodriguez, Santosh Kumar, Vishal Sharma, Gridsada Phanomchoeng, Ratchatin Chancharoen

**Affiliations:** 1 Department of Electrical Engineering, Wireless Communication Ecosystem Research Unit, Chulalongkorn University, Bangkok, Thailand; 2 Department of Electrical Engineering, University of Central Punjab, Lahore, Pakistan; 3 Department of Electronics and Communication Engineering, Guru Nanak Dev University, Amritsar, India; 4 Department of Electrical Engineering, College of Engineering, Taif University, Taif, Saudi Arabia; 5 College of Computer Engineering and Sciences, Prince Sattam Bin Abdulaziz University, Alkharj, Saudi Arabia; 6 Department of Computer Sciences, Federal University of Lavras, Lavras, Minas Gerais, Brazil; 7 School of Physics Science and Information Technology, Liaocheng University, Liaocheng, China; 8 Aston Institute of Photonics & Technologies, Aston University, Brimingham, United Kingdom; 9 Department of Mechanical Engineering, Chulalongkorn University, Bangkok, Thailand; Rutgers University Newark, UNITED STATES

## Abstract

Autonomous vehicles are regarded as future transport mechanisms that drive the vehicles without the need of drivers. The photonic-based radar technology is a promising candidate for delivering attractive applications to autonomous vehicles such as self-parking assistance, navigation, recognition of traffic environment, etc. Alternatively, microwave radars are not able to meet the demand of next-generation autonomous vehicles due to its limited bandwidth availability. Moreover, the performance of microwave radars is limited by atmospheric fluctuation which causes severe attenuation at higher frequencies. In this work, we have developed coherent-based frequency-modulated photonic radar to detect target locations with longer distance. Furthermore, the performance of the proposed photonic radar is investigated under the impact of various atmospheric weather conditions, particularly fog and rain. The reported results show the achievement of significant signal to noise ratio (SNR) and received power of reflected echoes from the target for the proposed photonic radar under the influence of bad weather conditions. Moreover, a conventional radar is designed to establish the effectiveness of the proposed photonic radar by considering similar parameters such as frequency and sweep time.

## 1 Introduction

**Fig 1:**
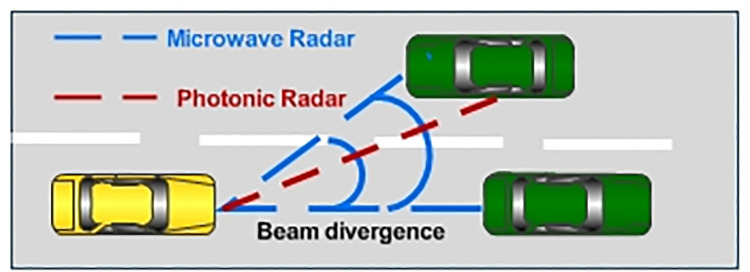
Microwave radar versus photonic radar in autonomous vehicle applications.

The last decade has witnessed remarkable growth in photonic radar applications for detecting long-distance targets, image classification and military surveillance, monitoring flood terrains and using in space applications [1]. Photonic radar is used as a primary method for detecting targets in all weather conditions. One of the key applications of photonic radar is in the Autonomous Vehicle (AV) industry due to its numerous advantages such as park assistance, lane detection, monitoring of blind spots and detection of multiple targets [2]. Autonomous cars can be integrated with photonic radars to avoid collision as well as to detect various signboards, pedestrians, trees, buildings, etc. Photonic radars can extract information from targets (such as speed, image, distance and altitude) by modulating radio frequency on optical signal which can be transmitted into free space by an optical transmitter [3, 4]. It can then collect the reflected signal (echoes) with the help of a receiver. These reflected signals are further processed to extract the required information. On the other hand, microwave radars which use radiation of radio frequencies highly suffer from low bandwidth, low speed and poor resolutions [5–7]. This makes the conventional microwave radars not an appropriate choice for the AV industry. Fig 1 shows the pictorial representation of microwave radar and photonic radar in autonomous driving vehicle applications. The beam divergence (which is defined as ratio of wavelength and aperture diameter of transmitter) for microwave radar is typically large which makes it difficult to differentiate between two cars. Whereas photonic radar has low beam divergence due to narrow linewidth offered by the operating laser which makes it an excellent choice for autonomous vehicles [8].

Moreover, photonic radar offers low input power requirements (≈<20W) as it is one of the important key requirements of autonomous vehicles due to availability of limited power supply from batteries [9–11]. In [11], authors have verified that power requirement of RADAR is higher than power requirement of Photonic radar. Further, millimetre band (75–77 GHz) is preferred by the radar industry in order to attain high resolutions [12]. However, transmission of microwave radar signals in millimetre band suffers from the atmospheric fluctuations which confine the radar’s detection ability [13, 14]. These atmospheric fluctuations can increase signal attenuation which can degrade the reception of signals through the atmosphere. Other atmospheric turbulences such as rainfall, fog, dust, etc. can impact the signal transmission from microwave radar which further leads to degradation of its performance. On the other hand, millimeter bands transmitted over photonic radar offers less environmental deterioration as signal is transmitted over light beams. Many researchers have calculated the impact of atmospheric turbulences on the performance of photonic radars. For achieving long-range target detection, frequency band selection in radars plays a very important role. S-band (2-4GHz) has strong immunity against atmospheric attenuations whereas X-band (8–12 GHz) has the advantage of narrow beams for tracking the targets [15]. In 2016 [16], authors have proposed photonic transceiver which can transmit and detect S and X band signals with the help of low sample rate-based analog to digital converter. In another work [17], authors have proposed linear frequency modulated continuous wave (LFMCW) based photonic radar for generating and detecting S and X band signals. Similarly, Ka-band (27–40 GHz) suffers high attenuation under atmospheric attenuations and hence can be used only for short-range applications such as in airports [18]. Range and velocity resolutions can be improved further by using 77 GHz optical RF-LFM (Radio Frequency-Linear Frequency Modulation) signals to realize frequency modulated continuous wavelength based photonic radar (FMCW-PHRAD) along with long optical pulses and low peak power requirements [19, 20]. AV radars can be operated at the frequency bands of 24 GHz and 77 GHz. This can help a frequency band of 77 GHz (≈4 GHz) achieve a higher bandwidth than a 24 GHz band (≈200 MHz) due to which light-based radar manufacturers prefer it. The higher bandwidth further improves range and velocity resolutions for detecting close-spaced targets and radar accuracy of long-range targets. In 2018 [4], authors have demonstrated that with the bandwidth of 1 GHz or more, the resolutions of detected target are significantly improved as compared to lower bandwidth range (≈200 MHz). In 2019 [21], authors have presented algorithm for detecting the moving targets with the help of 77 GHz automotive radar. In this work, authors have used constant false alarm rate (CFAR) and adjustable coefficient by using the Fast Fourier Transform (FFT) algorithm technique. This method has low alarm false rate as compared to previous methods.

In this work, we have designed a coherent FMCW-PHRAD by modulating 77 GHz frequency with the bandwidth of 600 MHz to realize the application for autonomous cars. The modelling of transmitter is carried out by using simulation software OptiSystem^TM^ whereas free space link is modelled by using the MATLAB^TM^ software. The remainder of the paper is structured as follows: Section II presents the working principle and system modelling whereas section III describes the observations and discussion of the results. The conclusion of this work is presented in section IV.

### 2 Working principle and system modeling

**Fig 2:**
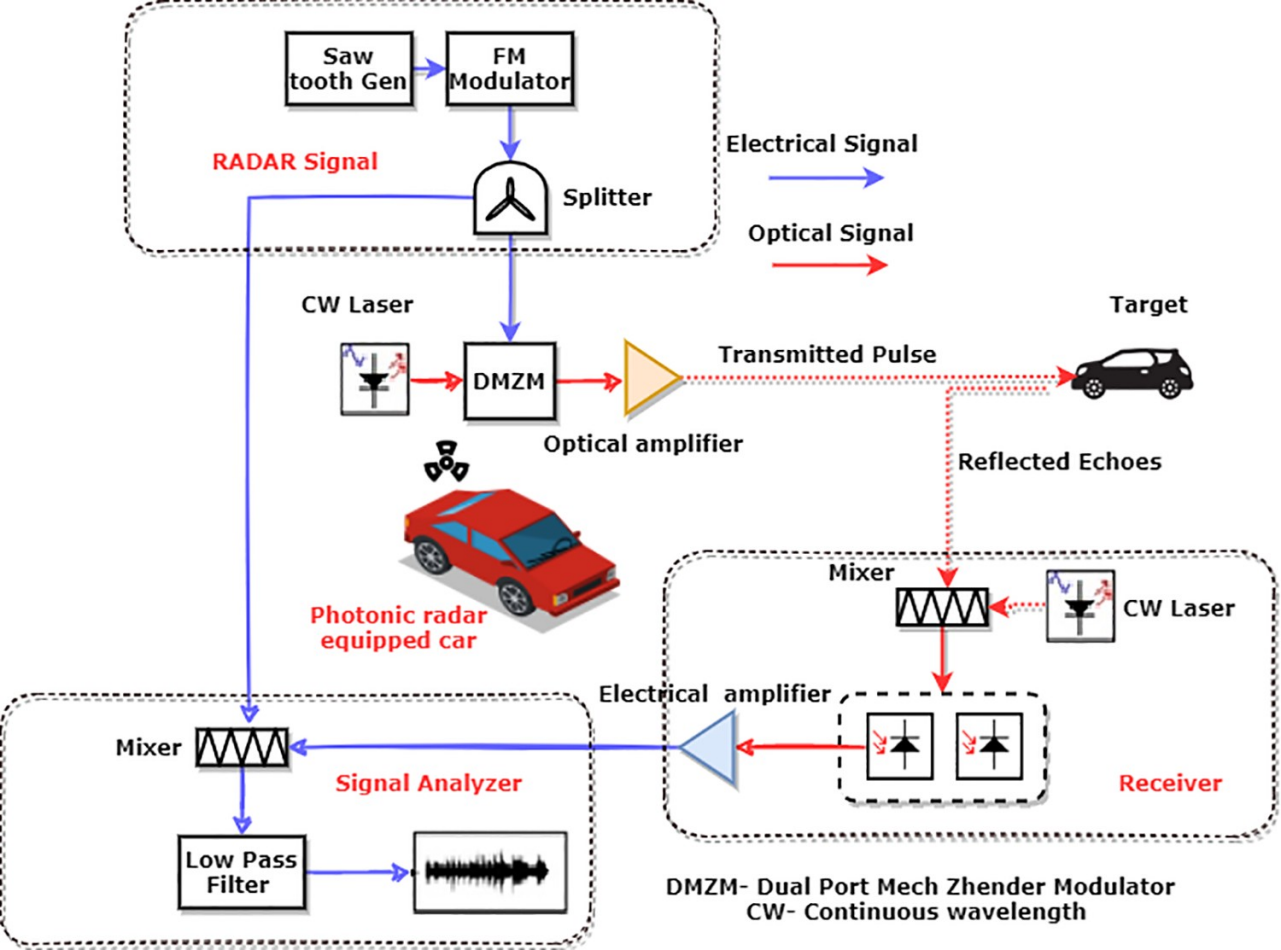
Proposed 77 GHz FMCW photonic radar.

Fig 2 demonstrates the configuration of the proposed photonic radar in coherent detection topology which uses the FMCW technique as autonomous cars have the requirement of low input power and compact size. FMCW-based photonic radar fulfils all the requirements of autonomous cars.

A saw tooth signal is modulated by using frequency modulator with the centre frequency of 77 GHz and bandwidth of 600 MHz to produce low-frequency radio frequency (RF) signal. The range frequency *f*_*R*_ of the target can be calculated as follows [22, 23]:

$$f_{R}=\frac{2\times B\times R}{T_{s}\times c}$$ (1)

where, *B* is the bandwidth, *R* refers to the distance, *c* the speed of light and *T*_*s*_ refers to the sweep time.

This radio frequency signal is split into two parts, one part is for modulating over optical carrier and the other part is mixed with the received signal from the target in order to recover the detected radar signal. The 77 GHz RF signal with 600 MHz bandwidth is modulated over an optical carrier with the help of dual-port Mech Zhender modulator (DMZM). DMZM is basically an intensity modulator which works on the principle of interferometric effect. A continuous wave laser with a centre frequency of 1550 nm and linewidth of 100 KHz is used to drive the MZM modulator. The power of laser is set to 100 mW which satisfies the minimum requirement of power for autonomous cars. DMZM operates in the configuration of null transmission in order to attain coherent detection. It contains two 3 dB couplers which are connected by two equal-length waveguides. With the effect of electro-optic, the refractive indexes of waveguides can be controlled by applying an external voltage which leads to suppression of higher order sidebands. The switching bias voltage and RF voltage of DMZM are set to 4*v* whereas bias voltage of 1V and -1V is applied to the two arms of the DMZM modulator. Thus, the modulated signal power *E(t)* at the output of DMZM is expressed as Eq 2 [23, 24]:

$$E(t)=\frac{E_{l}}{10^{\frac{IL}{20}}}[\gamma .e^{\left(-i\pi t\frac{v2(t)}{V_{\pi RF}}+i\pi t\frac{vbias2(t)}{V_{\pi DC}}\right)}+(1-\gamma ).e^{\left(-i\pi t\frac{v1(t)}{V_{\pi RF}}+i\pi t\frac{vbias1(t)}{V_{\pi DC}}\right)}]$$ (2)

where *E*_*l*_ is the laser input power, *v*_*1*_*(t)* and *v*_*2*_*(t)* are voltage values applied at the first and second arm of the modulator respectively, *v*_*bias1*_*(t)* and *v*_*bias2*_*(t)* are bias voltage values applied at the first and second arm of the modulator respectively, *γ* is the power splitting ratio [$\gamma =\frac{1}{2}(1-\frac{1}{\sqrt{er}})$; where *er = $10\frac{ER}{10}$. ER* is the extinction ratio], *IL* is the insertion-loss, *V*_*πRF*_ refers to the switching modulation voltage and *V*_*πDC*_ refers to the switching bias voltage. The output of DMZM is amplified by using flat gain optical amplifier with a gain of 20 dB and a noise power of 5 dB. The output gain of the optical amplifier is expressed as [25]:

$$G=\frac{P_{out}-P_{ASE}}{P_{Sin}}$$ (3)

where *P*_*out*_ is the output power, *P*_*Sin*_ is the input power and *P*_*ASE*_ is the generated and amplified spontaneous emission. The optical amplified signal is projected towards the target via free space channel through telescope which has the aperture diameter of 5 cm. The free space channel is modelled in the MATLAB^TM^ software with the help of *Phase array* toolbox. The visibility of less than <100m is considered to be hazardous for autonomous vehicles. Atmospheric turbulences, particularly rain and fog, are considered as the main challenges for autonomous vehicles which can degrade the performance of photonic radars especially in mmW band. However, attenuation caused by rain is relatively less as compared to fog due to small wavelength of optical signal as compared to rain droplet. The rain attenuation *A*_*rain*_ can be calculated as follows [26]:

$$A_{rain}=k.R_{o}^{\alpha }$$ (4)

where rainfall rate is denoted by *R*_*o*_ in mm/hr, *k* and *α* are power law factors that depend upon variables such as droplet size, frequency and temperature and can be computed using Marshall–Palmer distribution [27, 28]. Thus, the value of attenuation is considered as 6.9 dB/km, 4.6 dB/km and 2 dB/km for light rain, average rain and strong rain respectively [29]. Similarly, for computing the attenuation of fog, *Mie scattering* empirical model is used which is expressed as follows:

$$\beta (\lambda )=\frac{3.91}{V}{\left(\frac{\lambda }{550}\right)}^{-\rho }$$ (5)

where *λ* refers to the operating wavelength of the laser, *V* the visibility (kms) and *ρ* refers to the size distribution coefficient of scattering. The values for parameter visibility *V* are considered as 200 m for heavy fog, 500 m for moderate fog and 750 m for low fog conditions as per the ITU standards [29]. Thus, from the Eq (5), the attenuation for heavy fog is computed as 70 dB/km, 28.9 dB/km for moderate fog and 18.3 dB/km for light fog. The echoes reflected from the target are denoted with the help of receiver which consist of balance PIN photo diodes. In order to attain the heterodyne coherent detection, same CW laser signal, as used in transmitter side, should be mixed with the reflected echoes from the target as described in Fig 2.

The received power of echo signal *P*_*r*_ is calculated as:

$$P_{r}=\left\{ \begin{array}{l} P_{t}\frac{\rho_{t}D^{2}\tau_{opt}\tau_{atm}^{2}}{{4R}^{2}}\;for\;extended\;target\\ P_{t}\frac{\rho_{t}{A_{t}D}^{2}\tau_{opt}\tau_{atm}^{2}}{{4R}^{2}A_{ill}}\;for\;any\;target \end{array}\right.$$ (6)

where *D* is the receiver aperture diameter, *ρ*_*t*_ is the target reflectivity, *A*_*t*_ is the target area, *τ*_*opt*_ is the transmission loss in optical domain, *τ*_*atm*_ is the atmospheric loss factor, *A*_*ill*_ is the illuminated area at target and *R* is the target range. The receiver has two PINs and it recovers the baseband (radar RF) signal followed by the electrical subtractor. The incident optical electrical field *E*_*pd*1_ at photodetector 1 is given by [23]:

$$E_{pd1}=\frac{1}{\sqrt{2}}[E_{lo}(t)+jE_{ref}(t)]$$ (7)

whereas incident optical electrical field *E*_*pd*2_ at photodetector 2 is given by:

$$E_{pd2}=\frac{1}{\sqrt{2}}[E_{lo}(t)+E_{ref}(t)]$$ (8)

In the Eq (8) and (9), *E*_*lo*_(*t*) is expressed as below:

$$E_{lo}(t)=\sqrt{P_{lo}}e^{j(\omega_{o}(t)+\theta_{o}(t))}$$ (9)

where *θ*_*o*_(*t*) is the phase variation of signal (CW laser) and *P*_*lo*_ is the optical power (CW laser) and *E*_*ref*_(*t*) is the incident optical field from the target (reflected echoes) which can be expressed as [23]:

$$E_{ref}(t)=\sqrt{P_{r}}\;\cos[2\pi f_{start}(t-\tau )+\frac{\pi B}{T_{m}}{\left(t-\tau \right)}^{2}].e^{\left(j(\omega_{o}-\omega_{d})t+\theta_{o(t)}\right)}$$ (10)

where τ is the propagation delay given as *τ = 2 × R/c* (*R* refers to the range and *c* refers to the speed of light), *B* is the modulation bandwidth and *T*_*m*_ is the time duration. When the range is 750 m, delay time is calculated as 5 μsec at 200 kHz of pulse repetition frequency (PRF). The output current of the balanced photodetector with responsivity ℜ after using low pass filter is expressed as [23]:

$$i_{ph}(t)=2\times R\times \sqrt{P_{lo}\times P_{r}}\;\cos\;[2\pi f_{start}(t-\tau )+\frac{\pi \beta }{T_{m}}{\left(t-\tau \right)}^{2}]\sin{[\omega }_{d}(t)+(\theta_{o(t)}-\theta_{lo(t)})$$ (11)

In order to attain the beat signal ((*S*_*b*_(*t*)), the output from the balanced photodetector is mixed with radar RF signal and then passed through low pass filter which also determines the range frequency (*f*_*r*_) as well as doppler frequency (*f*_*d*_). *S*_*b*_(*t*) can be expressed as follows [23]:

$$S_{b}(t)=R\times A_{lo}\times \sqrt{P_{lo}\times P_{r}}\;\cos\;[2\pi f_{start}\tau +\frac{\pi \beta }{T_{m}}{\left(\tau \right)}^{2}+2\pi f_{r}(t)]\;\sin{[\omega }_{d}(t)+(\theta_{o(t)}-\theta_{lo(t)})]$$ (12)

where (*f*_*r*_) is the range frequency (Eq 1) and *A*_*lo*_ is the amplitude of radar RF signal. The other parameters considered for modelling the proposed coherent detection-based photonic radar are mentioned in Table 1.

**Table 1:** Modeling parameters for proposed photonic radar.

Component	Parameters	Value
Continuous wavelength Laser	Wavelength	1550 *nm*
	Linewidth	100 KHz
	Power	0.1 W
Dual Port Mechzender modulator (DMZM)	Extinction ratio	30 dB
	Switching bias voltage	4 V
	Switching RF voltage	4 V
	Bias Voltage	+1 V, -1 V
Simulation window	Sweep time	10 μs, 20 μs and 30 μs
	No of Samples	8192
	Delay time	5 μs
Photodetector (PIN)	Responsivity	1 A/W
	Dark current	1 nA
	Thermal noise bandwidth	410 MHz
	Absolute temperature	290 K
	Load resistance	50 *Ω*
	Shot noise bandwidth	410 MHz

## 3 Observations and discussions

**Fig 3:**
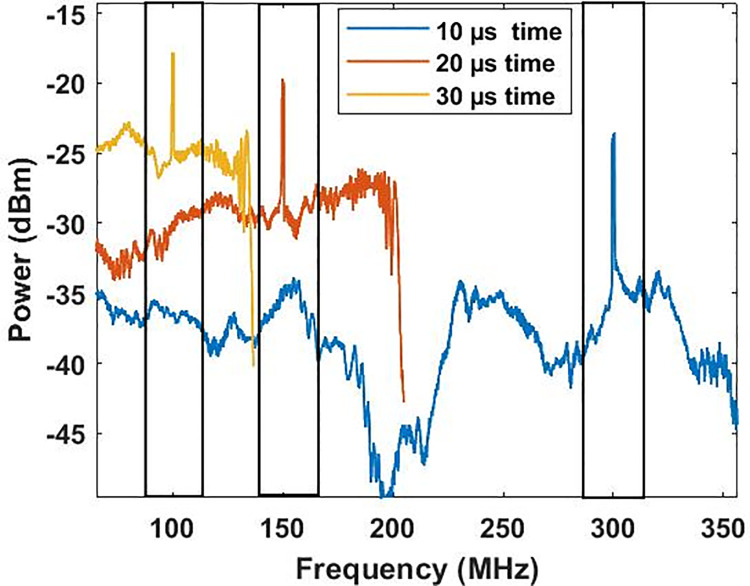
Reflected echoes from the target at 10 μs, 20 μs and 30 μs time sweep.

A comprehensive discussion is demonstrated in this section that describes the results obtained from the modelling of the proposed coherent detection-based photonic radar. The scintillations in the free space channel are assumed to be in ideal condition. A total number of 8192 samples are considered in the modelling. The detection of reflected echoes from the target located at the range of 750 m is presented in Fig 3. The atmospheric conditions are assumed to be clear weather conditions. In this condition, the peak of reflected echoes is detected at 300 MHz when the sweep time is set to 10 μs with the power threshold of -24 dBm. Similarly, when the sweep time is shifted to 20*μs*, the reflected echoes are detected at the range frequency (*f*_*R*_) of 150*MHz* with a threshold power of -19 dBm. The reflected echo frequency changes to 100 MHz with a threshold power of -17 dBm when the sweep time changes to 30 μs. This satisfies the theoretical Eq (1).

**Fig 4:**
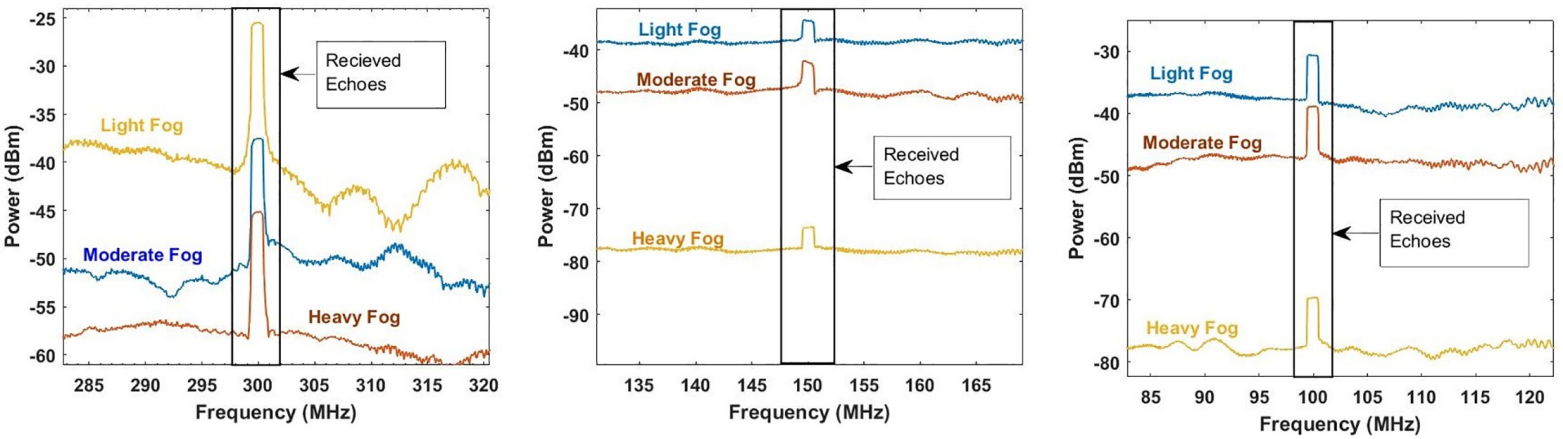
Fog analysis on reflected echoes from the target at sweep time of (a) 10 time sweep μs, (b) 20 μs and (c) 30 μs.

**Fig 5:**
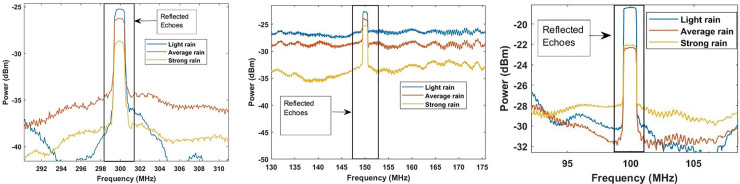
Impact of rain on reflected echoes from the target at sweep time of (a) 10 time sweep *μs*, (b) 20*μs* and (c) 30*μs*.

The effect of adverse weather turbulence conditions, particularly rain and fog, in the reflected echoes are presented in Figs 4 and 5, respectively.

Fig 4 determines the impact of fog (low, medium and heavy based on Eq (5) and (6)) on the performance of target detection by the proposed photonic radar at different time sweep. When the time sweep is set at 10 μs, the power of the reflected echo from the target is measured as -24 dBm with the range frequency of 300 MHz under light fog conditions. However, when the atmospheric fog changes to medium fog and heavy fog, the power of the reflected echo is measured as -38 dBm and -44 dBm, respectively. Similarly, at the time sweep range of 20 μs, the power of the reflected echo is computed as -36 dBm under light fog conditions. In the case of the atmospheric conditions changing into medium and heavy fog, it drastically reduces the power of the reflected echo which is computed as -42 dBm and -78 dBm respectively with the range frequency of 150 MHz. Similarly, when the time sweep is set at 30*μs*, the reflected echo is detected with the power of -32 dBm, -39 dBm and -70 dBm under the impact of low, medium and heavy fog conditions respectively with the range frequency of 100 MHz.

Fig 5 shows the impact of rainfall (Eq 4) on reflected echoes from the target. Under the influence of light rain, average rain and heavy rain at the sweep time of 10 μs, the reflected echo is detected with the power of -26 dBm, -27 dBm and -29 dBm, respectively. When the sweep time further changes to 20*μs*, the reflected echoes under the impact of light rain, average rain and heavy rain are detected with the power of -23 dBm, -24 dBm and -25 dBm, respectively. Furthermore, when the sweep time changes to 30 μs, the reflected echo is detected with the power of -19 dBm, -22 dBm and -23 dBm under the influence of light rain, average rain and heavy rain, respectively. Under the impact of fog and rain, the signal to noise ratio (SNR) is used for measuring the performance of the proposed photonic radar.

**Fig 6:**
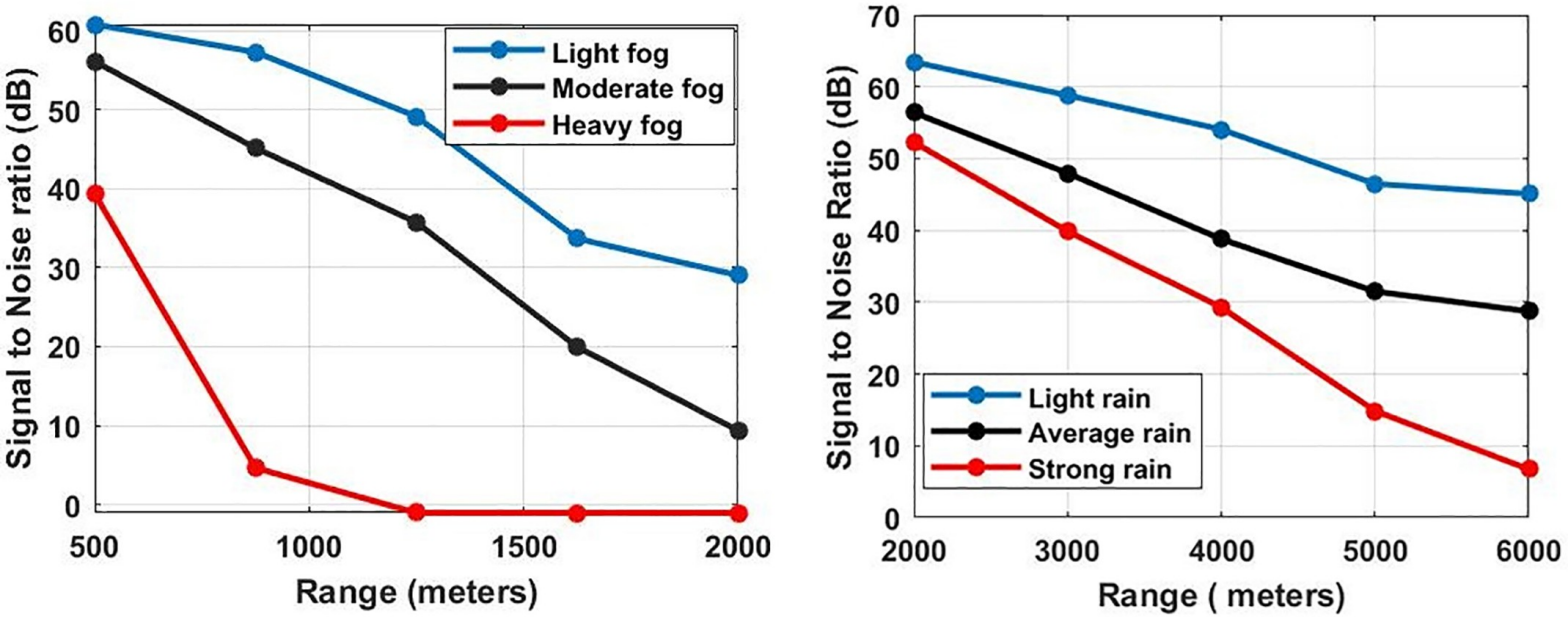
Evaluation of SNR under the impact of atmospheric attenuations at sweep time = 30 *μs* (a) Fog analysis (b) Rain analysis.

The SNR for the coherent heterodyne detection scheme can be measured as [23]:

$$SNR\;(heterodyne)=\frac{R^{2}P_{lo}\;P_{r}}{2qRP_{lo}B_{rx}+\frac{4k_{b}T_{r}B_{rx}}{R_{L}}}$$ (13)

where ℜ responsivity of the photodetector is 1*A/w*, *B*_*rx*_ is the thermal bandwidth of photodetector which is 410*MHz*, *T*_*r*_ is the absolute temperature of photodetector and it is equal to 290 K and *R*_*L*_ is the load resistance which is set at 50 Ω. Fig 6 shows the measured SNR under the impact of atmospheric attenuations (fog and rain).

When the weather condition changes to light fog, the SNR of the reflected echo is measured as 30 dB with the target distance capability of 2000 m and when there is moderate fog, the SNR of the reflected echo reduces to 10 dB with the same distance. In this case, target distance reduces to 1400 m to achieve the SNR of 30 dB. Furthermore, when the weather condition changes to heavy fog, the target distance reduces to 600 m only to achieve the SNR of 30 dB. Likewise, the impact of rain is less severe to the performance of radar as compared to fog. In the case of light rain, the reflected echo achieved 45 dB SNR at the target distance of 6000 m whereas under the influence of average rain, SNR reduced to 20 dB at the same distance. In the case of heavy rain, SNR reduced to 9 dB only at 6000 m of the target distance. To demonstrate the feasibility of the proposed FMCW photonic radar (sweep time = 30μs), the work was further extended by comparing with the conventional FMCW radar. A car moving at a speed of 19.44 m/s usually installs the conventional FMCW radar. This work also reported two scenarios for the FMCW radar equipped on moving cars. In the first scenario, the speed of the target car is assumed to be same with the FMCW radar-equipped car (detection) with the speed of 19.44 m/s. Therefore, the relative velocity of the target car is 0km/h as with respect to photonic radar equipped vehicle whereas in the second scenario the speed of the target car is different (lower) than the FMCW-equipped car. In case of both scenarios, the distance from the target car to the photonic radar is assumed as 50 m.

**Fig 7:**
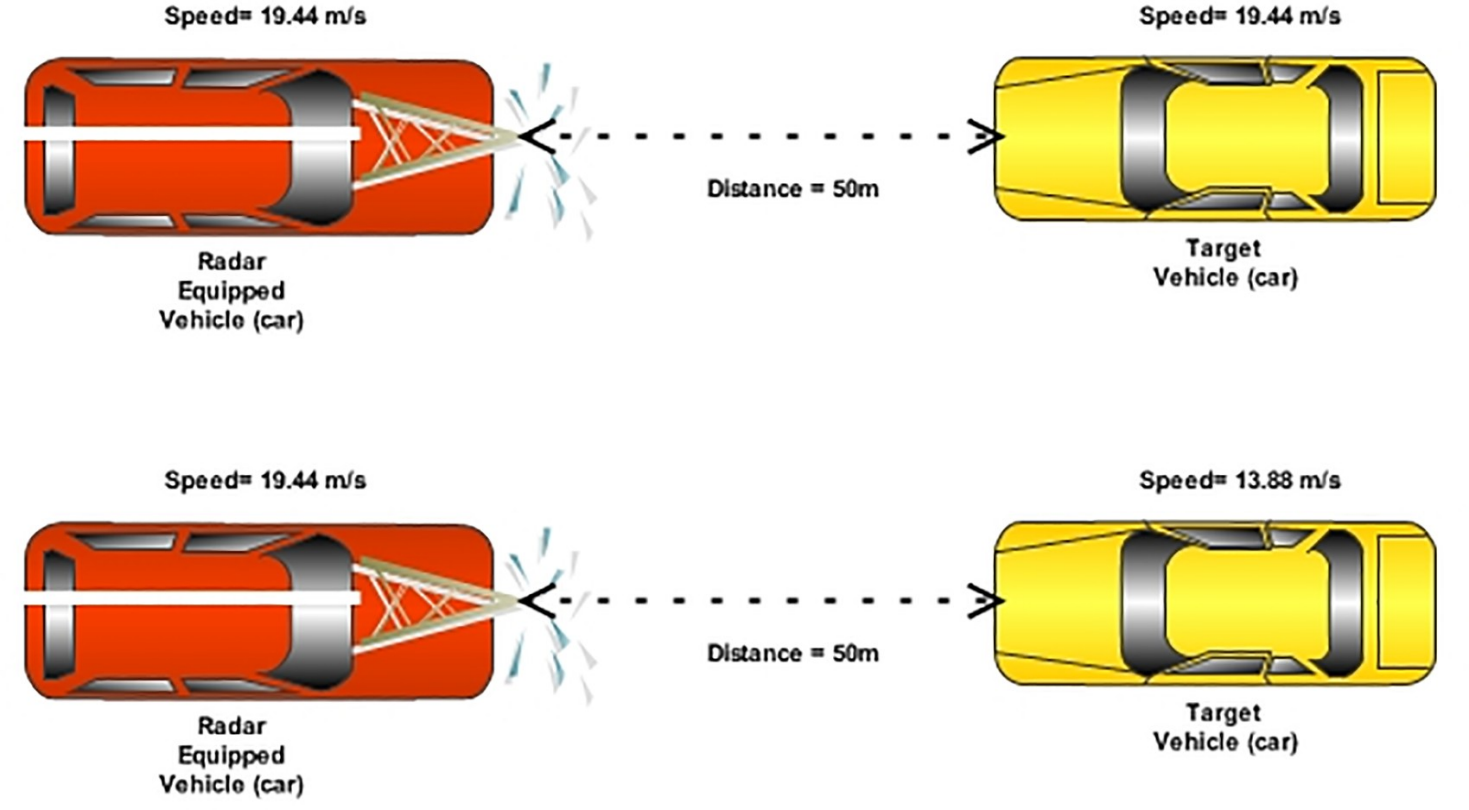
Pictorial representation of FMCW conventional radar (a) scenario 1 (b) scenario 2.

Fig 7 shows the representation of two scenarios. In the first scenario, the speed of the target car and the photonic radar-equipped vehicle is 19.44 m/s each. Therefore, the relative velocity of the target car is 0 m/s as with respect to the photonic radar-equipped vehicle. In case of both scenarios, the distance from the target car to the photonic radar is assumed as 50 m.

**Fig 8:**
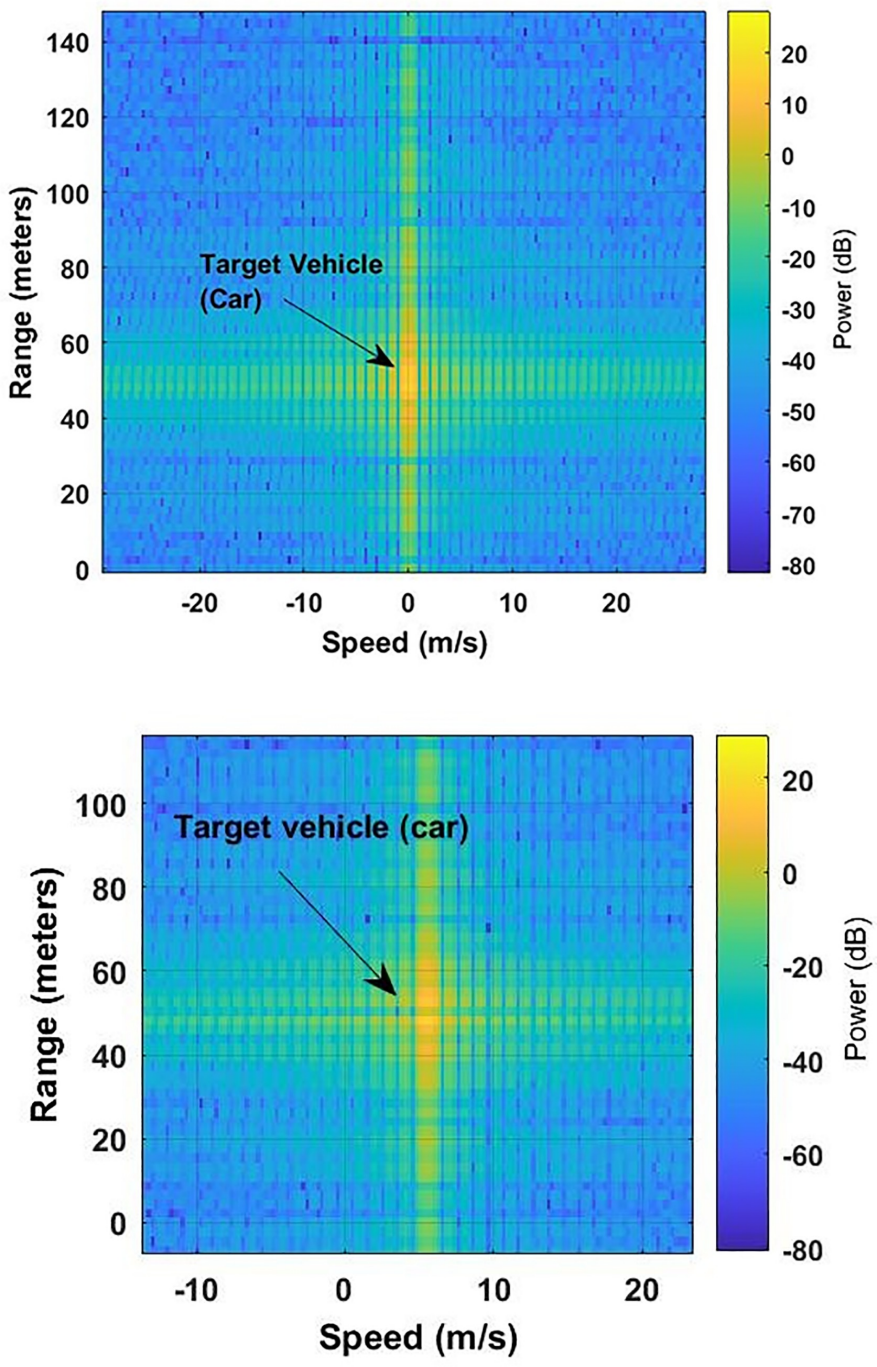
Radar range–speed response pattern (a) scenario 1 (b) scenario 2.

The maximum speed of the target vehicle and the FMCW radar-equipped vehicle is assumed as 41.66 m/s. To compare with the proposed photonic radar, the sweep time of the conventional FMCW radar is set to 30 μs with the bandwidth of 600 MHz. Fig 8A) shows the detection of target by computing radar range response with respect to relative velocity. The target vehicle is detected at 50 m from the photonic radar-equipped vehicle. In the second scenario, the speed of the target vehicle is 13.88 m/s, hence the relative velocity is 5.56 m/s with respect to the FMCW radar-equipped vehicle. Fig 8(B) shows the detection of the target vehicle which moved with the relative velocity of 5.56 m/s towards the radar-equipped vehicle.

## 4 Conclusion

In this work, we have designed FMCW-based coherent photonic radar for autonomous vehicle applications. The free space link is modelled in the MATLAB^TM^ software. The observation shows the successful detection of target at different sweep times of 10 μs, 20 μs and 30 μs. However, the reflected echo has computed the highest power at 30 μs as compared to 10 μs and 20 μs. Furthermore, the effects of various atmospheric weather conditions particularly rain and fog are investigated on the performance of the proposed FMCW-based coherent photonic radar. To achieve the SNR of 30 dB, the target range prolongs to 2000 m for light fog conditions and 1400m for moderate fog conditions whereas for heavy fog conditions, target range prolongs to only 600 m. Similarly, under the impacts of low rain, average rain and heavy rain, the target range prolongs to 6000 m with the SNRs of 45 dB, 20 dB and 9 dB, respectively. Moreover, the FMCW-based conventional radar is designed and compared with the FMCW-based photonic radar to establish the effectiveness of the proposed coherent FMCW-based photonic radar by considering same parameters. The reported range-speed patterns of the radar show the successful detection of moving target vehicles. In future, this work is further extended by real-time experiments with complex traffic conditions.

## References

[1] Pan S. and Zhang Y., "Microwave Photonic Radars," *Journal of Lightwave Technology*, vol. 38, pp. 5450–5484, 2020/10/01 2020.

[2] Shi J.-W., Guo J.-I., Kagami M., Suni P., and Ziemann O., "Photonic technologies for autonomous cars: feature introduction," *Optics Express*, vol. 27, pp. 7627–7628, 2019/03/04 2019. doi: 10.1364/OE.27.007627 30876324

[3] Serafino G., Scotti F., Lembo L., Hussain B., Porzi C., Malacarne A., et al., "Toward a New Generation of Radar Systems Based on Microwave Photonic Technologies," *Journal of Lightwave Technology*, vol. 37, pp. 643–650, 2019/01/15 2019.

[4] Serafino G., Amato F., Maresca S., Lembo L., Ghelfi P., and Bogoni A., "Photonic approach for on-board and ground radars in automotive applications," *IET Radar, Sonar & Navigation*, vol. 12, pp. 1179–1186, 2018.

[5] Dudek M., Nasr I., Bozsik G., Hamouda M., Kissinger D., and Fischer G., "System analysis of a phased-array radar applying adaptive beam-control for future automotive safety applications," *IEEE Transactions on Vehicular Technology*, vol. 64, pp. 34–47, 2014.

[6] Ghelfi P., Laghezza F., Scotti F., Serafino G., Pinna S., Onori D., et al., "Photonics in Radar Systems: RF Integration for State-of-the-Art Functionality," *IEEE Microwave Magazine*, vol. 16, pp. 74–83, 2015.

[7] Sharma V., Kbashi H. J., and Sergeyev S., "MIMO-employed coherent photonic-radar (MIMO-Co-PHRAD) for detection and ranging," *Wireless Networks*, vol. 27, pp. 2549–2558, 2021/05/01 2021.

[8] Piatek S. and Li J., "A photonics guide to the autonomous vehicle market," *Laser Focus World*, vol. 53, pp. 28–31, 2017.

[9] Molebny V., "Nick-named laser radars," *Advanced Optical Technologies*, vol. 8, pp. 425–435, 2019.

[10] Ghelfi P., Laghezza F., Scotti F., Serafino G., Capria A., Pinna S., et al., "A fully photonics-based coherent radar system," *Nature*, vol. 507, pp. 341–345, 2014. doi: 10.1038/nature13078 24646997

[11] Baxter J. A., Merced D. A., Costinett D. J., Tolbert L. M., and Ozpineci B., "Review of Electrical Architectures and Power Requirements for Automated Vehicles," in *2018 IEEE Transportation Electrification Conference and Expo (ITEC)*, 2018, pp. 944–949.

[12] Steeg M., Al Assad A., and Stöhr A., "All photonic radar system based on laser frequency sweeping and leaky-wave antennas," in *2018 International Topical Meeting on Microwave Photonics (MWP)*, 2018, pp. 1–4.

[13] Shrestha S. and Choi D.-Y., "Rain attenuation statistics over millimeter wave bands in South Korea," *Journal of Atmospheric and Solar-Terrestrial Physics*, vol. 152, pp. 1–10, 2017.

[14] Siles G. A., Riera J. M., and Garcia-del-Pino P., "Atmospheric attenuation in wireless communication systems at millimeter and THz frequencies [wireless corner]," *IEEE Antennas and Propagation Magazine*, vol. 57, pp. 48–61, 2015.

[15] Richards M. A., Scheer J., Holm W. A., and Melvin W. L., "*Principles of modern radar*," 2010.

[16] Scotti F., Bogoni A., Laghezza F., and Onori D., "Tracking of a naval target with a dual-band photonic-based coherent radar system," in *2016 IEEE Radar Conference (RadarConf)*, 2016, pp. 1–4.

[17] Meng Z., Li J., Yin C., Fan Y., Yin F., Zhou Y., et al., "Dual-band dechirping LFMCW radar receiver with high image rejection using microwave photonic I/Q mixer," *Optics express*, vol. 25, pp. 22055–22065, 2017. doi: 10.1364/OE.25.022055 29041495

[18] Scotti F., Laghezza F., Ghelfi P., and Bogoni A., "Multi-band software-defined coherent radar based on a single photonic transceiver," *IEEE Transactions on Microwave Theory and Techniques*, vol. 63, pp. 546–552, 2015.

[19] Mao X., Inoue D., Kato S., and Kagami M., "Amplitude-modulated laser radar for range and speed measurement in car applications," *IEEE Transactions on Intelligent Transportation Systems*, vol. 13, pp. 408–413, 2011.

[20] Harris M., Young R. I., Köpp F., Dolfi A., and Cariou J.-P., "Wake vortex detection and monitoring," *Aerospace Science and Technology*, vol. 6, pp. 325–331, 2002/09/01/ 2002.

[21] Mabrouk M., Abdullah H. H., Hussein K., and Hussein A. H., "A Novel Algorithm for Moving/Fixed Target Discrimination in 77 GHz Automotive Radars," in *2019 Photonics & Electromagnetics Research Symposium-Fall (PIERS-Fall)*, 2019, pp. 128–133.

[22] Gao S. and Hui R., "Frequency-modulated continuous-wave lidar using I/Q modulator for simplified heterodyne detection," *Optics letters*, vol. 37, pp. 2022–2024, 2012. doi: 10.1364/OL.37.002022 22660108

[23] Elghandour A. H. and Ren C. D., "Modeling and comparative study of various detection techniques for FMCW LIDAR using optisystem," in *International Symposium on Photoelectronic Detection and Imaging 2013*: *Laser Sensing and Imaging and Applications*, 2013, p. 890529.

[24] Gultepe I., Tardif R., Michaelides S., Cermak J., Bott A., Bendix J., et al., "Fog research: A review of past achievements and future perspectives," *Pure and applied geophysics*, vol. 164, pp. 1121–1159, 2007.

[25] Okoshi T., "Exact noise-figure formulas for optical amplifiers and amplifier-fiber cascaded chains," in *IEEE/OSA Topical Meeting on Optical Amplifiers and their Applications*, 1990.

[26] Rashidi F., He J., and Chen L., "Spectrum slicing WDM for FSO communication systems under the heavy rain weather," *Optics Communications*, vol. 387, pp. 296–302, 2017.

[27] Yangang L., "Statistical theory of the Marshall-Palmer distribution of raindrops," *Atmospheric Environment*. *Part A*. *General Topics*, vol. 27, pp. 15–19, 1993.

[28] Olsen R., Rogers D. V., and Hodge D., "The aR b relation in the calculation of rain attenuation," *IEEE Transactions on antennas and propagation*, vol. 26, pp. 318–329, 1978.

[29] Awan M. S., Leitgeb E., Hillbrand B., Nadeem F., and Khan M., "Cloud attenuations for free-space optical links," in *2009 International Workshop on Satellite and Space Communications*, 2009, pp. 274–278.

