# Correction: Coherent detection-based photonic radar for autonomous vehicles under diverse weather conditions

**DOI:** 10.1371/journal.pone.0260799

**Published:** 2021-11-29

**Authors:** The PLOS ONE Staff

There is information missing from the Funding statement. The publisher apologizes for the error. The correct Funding statement is as follows: This research project is supported by the Second Century Fund (C2F), Chulalongkorn University, Thailand. This research work is also funded by TSRI Fund (CU_FRB640001_01_21_8). This project is also supported by Taif University Researchers supporting project number (TURSP-2020/228), Taif University, Taif, Saudi Arabia. The funders had no role in study design, data collection and analysis, decision to publish, or preparation of the manuscript.
